# Client-side versus server-side geographic data processing performance comparison: Data and code

**DOI:** 10.1016/j.dib.2019.104507

**Published:** 2019-09-13

**Authors:** Marcin Kulawiak

**Affiliations:** Department of Geoinformatics, Faculty of Electronics, Telecommunication and Informatics, Gdansk University of Technology, Poland

**Keywords:** Geoprocessing, Performance, Scalability, GIS, Web

## Abstract

The data and code presented in this article are related to the research article entitled “Analysis of Server-side and Client-side Web-GIS data processing methods on the example of JTS and JSTS using open data from OSM and Geoportal” (Kulawiak et al., 2019). The provided 12 datasets include multi-point and multi-polygon data of different scales and volumes, representing real-world geographic features. The datasets cover the area of Tricity in northern Poland as well as Polish Exclusive Economic Zone of the Baltic Sea. They have been converted to a common Spherical Mercator projection coordinate system (EPSG:3857) and consist of vector features without attributes. They are provided in the form of single GeoJSON files containing multi-feature objects which can be processed by client-side as well as server-side algorithms in a single request. The provided javascript code exemplifies the application of those datasets for measurement of client-side and server-side geoprocessing performance by using algorithms implemented as part of Java Topology Suite (JTS) and Javascript Topology Suite (JSTS). The combination of data and code samples constitutes a universal benchmark for investigative analysis of geographic data processing algorithms and their implementations in different software system architectures.

**Table:** Specifications Table

Subject area	*Computer science, Environmental science*
More specific subject area	*Design and Analysis Of Algorithms, Data processing, Geographic Information Systems, Computer System Architecture*
Type of data	*Vector features, computer code*
How data was acquired	*Vector features used in Datasets 1,2,4 and 6 have been obtained from OpenStreetMap, HELCOM and the Polish State Border Registry, which provide up-to-date geographic data which may be freely used, shared and modified. After cropping to the area of interest (Tricity in Northern Poland in the case of OSM datasets, Polish Special Economic Zone of the Baltic Sea in the case of the HELCOM datasets) the datasets have been reprojected into a common Spherical Mercator projection coordinate system. Moreover, in order to ensure that the datasets contain only relevant information, all original attributes have been removed. Dataset 3 has been produced using the JTS "Buffer" operation on the Tricity railway network polyline. Datasets 7,8,9 and 10 have been generated using the "random points" tool available in QGIS. Datasets 11 and 12 consist of pre-processed WPS queries which operate on datasets 1–10. Code samples contain sample Javascript code for executing geoprocessing operations on the datasets 1–10 using both JTS and JSTS.*
Data format	*Processed*
Experimental factors	*Datasets have been chosen* with the intention to represent real-world data geoprocessing scenarios involving various types of vector features (points as well as polygons). In order to provide a common reference for testing both processing performance and resource requirements of geoprocessing algorithms (both client-side and server-side), the datasets provide a good variability in size as well as number of features.
Experimental features	*Pre-processed set of building features in Tricity, Poland (dataset 1), Pre-processed set of building features in city of Gdynia, Poland (dataset 2), estimated area of train noise in Tricity (dataset 3), Polish Exclusive Economic Zone of Baltic Sea (dataset 4), pre-processed illegal oil discharges in Baltic Sea (dataset 5), pre-processed illegal oil discharges in the Polish Exclusive Economic Zone (dataset 6), generated set of 1000 point features in the Polish Exclusive Economic Zone (dataset 7), generated set of 10000 point features in the Polish Exclusive Economic Zone (dataset 8), generated set of 30000 point features in the Polish Exclusive Economic Zone (dataset 9), generated set of 50000 point features in the Polish Exclusive Economic Zone (dataset 10), collection of pre-generated WPS queries for performance testing (dataset 11), collection of pre-generated WPS queries for testing performance scaling (dataset 12), sample computer code for performing measurements (code samples)*
Data source location	*Tricity, Poland, Polish Exclusive Economic Zone of the Baltic Sea*
Data accessibility	*Data are available within the article.*
Related research article	Kulawiak, M., Dawidowicz, A., Pacholczyk, M.E. 2019. Analysis of Server-side and Client-side Web-GIS data processing methods on the example of JTS and JSTS using open data from OSM and Geoportal. Computers & Geosciences 129, pp. 26–37.

**Table:** 

**Value of the data** • The data represents an established and ready-to-use toolset for researchers studying the evolution of performance differences between server-side and client-side spatial data processing efficiency. • Consisting of real-world vector features of different size and complexity, the provided datasets will aid researchers in investigation and optimization of performance as well as memory consumption scaling of geoprocessing algorithms. • With provided code, data can be used for analysis of algorithm efficiency, discovery of potential performance issues, investigation of architectural differences etc. • Alongside an established and proven methodology, the data in this article enables reference comparison between different algorithm implementations. • Potential end users include researchers in the field of computer science (investigation of differences in performance of software architectures, algorithm optimization) as well as environmental science (design and verification of geoprocessing algorithms/methods).

## 1 Data

This article contains two types of data: vector features and computer code. They constitute three types of datasets:

1. Datasets created manually by the author (datasets 7,8,9,10, and Code samples, consisting of vector features and computer code),
2. Datasets obtained from open-license sources and processed by the author (datasets 1,2,4,5,6, consisting of vector features),
3. Data created by the author with the use of the above datasets (datasets 3,11,12, consisting of vector features and computer code).

The datasets of the 2nd type (those based on open data sources) consist of:

1. Vector polygons representing buildings in the Tricity, a metropolitan complex comprised of the cities of Gdynia, Sopot and Gdansk. Tricity is located in Northern Poland, on the shores of the Baltic Sea. The data originates from OpenStreetMap, which is made available under the Open Database License (ODbL) [2]. The data has been obtained through the conversion site operated by Geofabrik GmbH under the same license [3].
2. A vector polygon representing the borders of the Polish exclusive economic zone of the Baltic Sea. The data has been obtained from the Polish Centre of Geodesic and Cartographic Documentation under the CC-BY license [4].
3. Vector markers representing illegal oil discharges discovered by means of aerial surveillance in the Baltic Sea area between 1998 and 2015. The data has been obtained from the HELCOM Baltic Sea Environmental Monitoring database. The data is made available under the creative commons license CC-BY [5].

### 1.1 Vector feature datasets

The datasets containing vector features are described in detail in Table 1.

**Table 1:** Description of vector feature datasets.

Name	Description	Number of features	Size	Format	License
Dataset 1	Buildings in the Tricity.	64 582 polygons comprised of 463 942 points.	22963 KB	GeoJSON	ODbL 1.0
Dataset 2	Buildings in the City of Gdynia.	18 355 polygons comprised of 144 704 points.	7155 KB	GeoJSON	ODbL 1.0
Dataset 3	Simulated area of Tricity affected by railway noise.	1 polygon comprised of 10 708 points.	459 KB	GeoJSON	ODbL 1.0
Dataset 4	Boundary of the Polish exclusive economic zone.	1 polygon comprised of 943 points.	41 KB	GeoJSON	CC-BY
Dataset 5	Illegal oil discharges discovered in the Baltic Sea area between 1998 and 2015.	4337 points.	186 KB	GeoJSON	CC-BY
Dataset 6	Illegal oil discharges observed in the Polish exclusive economic zone, 1998–2015	357 points.	16 KB	GeoJSON	CC-BY
Dataset 7	Set of randomly distributed point features in the Polish exclusive economic zone.	1000 points.	44 KB	GeoJSON	CC-BY
Dataset 8	Set of randomly distributed point features in the Polish exclusive economic zone.	10 000 points	428 KB	GeoJSON	CC-BY
Dataset 9	Set of randomly distributed point features in the Polish exclusive economic zone.	30 000 points.	1283 KB	GeoJSON	CC-BY
Dataset 10	Set of randomly distributed point features in the Polish exclusive economic zone.	50 000 points.	2137 KB	GeoJSON	CC-BY

### 1.2 Computer code datasets

The datasets containing computer code are described below.

#### 1.2.1 Dataset 11

A collection of pre-generated Web Processing Service (WPS) queries for performance testing. The queries include the following operations:

• intersectionBalticZone - a WPS operation of intersection between Dataset 4 and Dataset 5. Represents a real-world application of intersection on relatively small datasets.
• intersectionGdyniaNoise - a WPS operation of intersection between Dataset 2 and Dataset 3. Represents a real-world application of intersection on medium-sized polygon datasets.
• intersectionTricityNoise - a WPS operation of intersection between Dataset 1 and Dataset 3. Represents a real-world application of intersection on a relatively large polygon dataset.
• bufferZone - a WPS operation of buffer to be performed on Dataset 6. Represents a real-world application of buffering a small dataset.
• bufferBaltic -a WPS operation of buffer to be performed on Dataset 5. Represents a real-world application of buffering a medium-sized dataset.

#### 1.2.2 Dataset 12

A collection of pre-generated WPS queries for testing performance scaling. The queries include the following operations:

• buffer1000 - a WPS buffer operation to be performed on Dataset 7. Meant for testing performance of buffering a small dataset.
• buffer10000 - a WPS buffer operation to be performed on Dataset 8. Meant for testing performance of buffering a medium-sized dataset.
• buffer30000 - a WPS buffer operation to be performed on Dataset 9. Meant for testing performance of buffering a medium-large dataset.
• buffer50000 - a WPS buffer operation to be performed on Dataset 10. Meant for testing performance of buffering a large dataset.

#### 1.2.3 Code samples

Sample javascript code which presents the methods of using the provided datasets for measuring data processing performance. The set includes functions for processing Datasets 1–10 with JSTS and OpenLayers, as well as code examples of using Datasets 11 and 12 to investigate server-side data processing with JTS.

## 2 Experimental design, materials and methods

The datasets presented here have been created as input data for a methodology of measuring the efficiency of processing the same data using identical algorithms on the side of the client and server. The proposed methodology involves comparing the performance of client-side processing of GeoJSON files using the Javascript Topology Suite (JSTS) [6] with the performance of server-side processing via Web Processing Service (WPS) requests to an instance of the Java Topology Suite (JTS) [7]. After their creation or acquisition, the vector data have been converted into a common Spherical Mercator projection coordinate system (EPSG:3857, also known as Web Mercator) using Quantum GIS. Because the feature collections were intended for testing the performance of geoprocessing operations in a web environment, they have been stripped of all attributes in order to ensure that only relevant information is processed. Following that, the vector datasets have been converted into single files in the open GeoJSON format. These files contain multi-feature objects which can be processed by JTS and JSTS in a single request. The GeoJSON files, which can be used to test the performance of JSTS, are available in Datasets 1–10.

The presented datasets are meant to be used with a set of performance tests which have been designed to reflect real-world data use scenarios. These use cases involve geoprocessing of spatial data for the purposes of researching hazards in marine as well as municipal environments.

The intended tests, which may be executed on the side of the client (via JSTS) as well as the server (via JTS) using the same algorithm, include [1]:

1. Intersection of a single polygon feature with a medium-sized point dataset - Meant to identify illegal oil spills in the area administered by Polish Marine Offices by the intersection between the polygon representing the Polish exclusive economic zone (Dataset 4) and the collection of illegal oil discharges in the Baltic Sea area (Dataset 5). The operation is provided in WPS form as part of Dataset 11.
2. Intersection of a single polygon feature with a medium-large polygon dataset - Intended to estimate hazards to citizen health by identifying residential buildings in the City of Gdynia (Dataset 2) whose residents may be exposed to high levels of railway noise by intersecting them with the Tricity train noise polygon (Dataset 3). The operation is provided in WPS form as part of Dataset 11.
3. Intersection of a single polygon feature with a large polygon dataset - Intended to estimate hazards to citizen health by identifying residential buildings in the Tricity (Dataset 1) whose residents may be exposed to high levels of railway noise by intersecting them with the Tricity train noise polygon (Dataset 3). The operation is provided in WPS form as part of Dataset 11.
4. Buffering a relatively small point dataset - This operation intends to approximate the environmental impact of oil spills in the sector administered by Polish Marine Offices by estimating the area contaminated by each spill. This is achieved by performing a buffer operation on the illegal oil discharges in the Polish exclusive economic zone (Dataset 6). The operation is provided in WPS form as part of Dataset 11.
5. Buffering a medium-sized point dataset - This operation is similar to the previous one, however it performs buffering of all illegal oil spills in the Baltic Sea area (Dataset 5). The operation is provided in WPS form as part of Dataset 11.
6. Buffering a 1000-point dataset - This operation performs buffering of 1000 randomly placed points in the Polish exclusive economic zone (Dataset 7). The operation is provided in WPS form as part of Dataset 12.
7. Buffering a 10 000-point dataset - This operation performs buffering of 10 000 randomly placed points in the Polish exclusive economic zone (Dataset 8). The operation is provided in WPS form as part of Dataset 12.
8. Buffering a 30 000-point dataset - This operation performs buffering of 30 000 randomly placed points in the Polish exclusive economic zone (Dataset 9). The operation is provided in WPS form as part of Dataset 12.
9. Buffering a 50 000-point dataset - This operation performs buffering of 50 000 randomly placed points in the Polish exclusive economic zone (Dataset 10). The operation is provided in WPS form as part of Dataset 12.

Operations 1–5 are meant to test processing performance of different geoprocessing algorithms on datasets of various sizes. Operations 6–9 are intended for testing performance scaling of the buffering operation under different data loads. The enclosed javascript code provides examples of executing tests 1–5 and 6–9 on the client side with the use of OpenLayers and JSTS. For server-side testing, the operations 1–5 and 6–9 have been implemented in the form of JTS WPS queries, which are available in Dataset 11 and Dataset 12, respectively. The queries may be executed on a GeoServer WPS instance via the provided javascript code. For detailed instructions regarding using the datasets with OpenLayers and GeoServer, see the attached code samples. Details regarding testing environment and methodology as well as reference test results may be found in [1].
